# Evolution of Treatment Regimens in Multiple Myeloma: A Social Network Analysis

**DOI:** 10.1371/journal.pone.0104555

**Published:** 2014-08-13

**Authors:** Helen Mahony, Athanasios Tsalatsanis, Ambuj Kumar, Benjamin Djulbegovic

**Affiliations:** 1 Department of Internal Medicine, Division of Evidence-Based Medicine & Health Outcomes Research, University of South Florida, Tampa, Florida, United States of America; 2 H. Lee Moffitt Cancer Center & Research Institute, Department of Hematology and Health Outcomes and Behavior, Tampa, Florida, United States of America; National Taiwan University, Taiwan

## Abstract

**Background:**

Randomized controlled trials (RCTs) are considered the gold standard for assessing the efficacy of new treatments compared to standard treatments. However, the reasoning behind treatment selection in RCTs is often unclear. Here, we focus on a cohort of RCTs in multiple myeloma (MM) to understand the patterns of competing treatment selections.

**Methods:**

We used social network analysis (SNA) to study relationships between treatment regimens in MM RCTs and to examine the topology of RCT treatment networks. All trials considering induction or autologous stem cell transplant among patients with MM were eligible for our analysis. Medline and abstracts from the annual proceedings of the American Society of Hematology and American Society for Clinical Oncology, as well as all references from relevant publications were searched. We extracted data on treatment regimens, year of publication, funding type, and number of patients enrolled. The SNA metrics used are related to node and network level centrality and to node positioning characterization.

**Results:**

135 RCTs enrolling a total of 36,869 patients were included. The density of the RCT network was low indicating little cohesion among treatments. Network Betweenness was also low signifying that the network does not facilitate exchange of information. The maximum geodesic distance was equal to 4, indicating that all connected treatments could reach each other in four “steps” within the same pathway of development. The distance between many important treatment regimens was greater than 1, indicating that no RCTs have compared these regimens.

**Conclusion:**

Our findings show that research programs in myeloma, which is a relatively small field, are surprisingly decentralized with a lack of connectivity among various research pathways. As a result there is much crucial research left unexplored. Using SNA to visually and analytically examine treatment networks prior to designing a clinical trial can lead to better designed studies.

## Introduction

Multiple myeloma (MM) is a hematological malignancy, which accounts for 1% of cancer deaths and 10% of all hematological malignancies in the US. [1] Several advances in the treatment of MM have been possible due to testing of newer agents in randomized controlled trials (RCTs). As a result of these advancements, MM has transformed into a chronic disease.

The first randomized controlled trial (RCT) in MM was published in 1966. [2] Since then over 300 RCTs have been published and nearly half have been conducted in an induction and transplant setting. [3] While there is a vast body of research involving RCTs in MM as well as systematic reviews and meta-analyses [4]–[7], there has been no formal assessment of patterns of treatment discoveries in the context of RCTs. That is, it is not known how a new treatment regimen makes its way through the translational cycle to be tested in an RCT. Understanding the process of this translational cycle, specifically in relation to the choice of new regimens for the treatment of MM in RCTs, is important and has several benefits. At the highest level, it can provide investigators interested in assessing the efficacy of similar treatment regimens with collaborative opportunities, thereby avoiding duplication of research efforts. Avoiding duplication can result in not only consolidation of research efforts and allocation of limited resources, but can also lead to averting patients from participating in trials where the answers to the treatment efficacy may already be known.

Using methodologies that provide analytical as well as visual representations of existing research in MM, prior to conducting new RCTs, could lead to better designed studies and enable researchers to address more relevant clinical and research questions. In this paper, we propose using social network analysis (SNA) to study the patterns of interactions between treatment regimens in RCTs, to identify potential limitations, and draw future research directions. SNA provides a “bird's-eye view” of the overall RCT universe, which enables us to examine relationships, directions, and importance of different treatment regimens in the network. Such an approach could allow industry and government to save valuable healthcare dollars by focusing resources on relevant research.

We have previously reported on a preliminary SNA on RCTs of autologous stem cell transplant (ASCT) and novel agents for MM [8]. Our analyses showed that research programs in myeloma, which is a relatively small field, were surprisingly decentralized and various research pathways suffered by lack of connectivity. We hypothesize that this finding is attributed to lack of interaction among researchers performing RCTs and the absence of policies that enforce consolidation of the totality of existing evidence prior to designing new RCTs. In this paper, we expand our initial analyses to include all RCTs of induction or ASCT among patients with MM and we illustrate a novel application of SNA to study the topology of RCT treatment networks.

## Methods

### Dataset

We searched MEDLINE for any relevant RCTs published until April 2012 using the searching strategies described by Haynes et al. [9] No limits of any kind were imposed. Abstracts from the American Society of Hematology and the American Society of Clinical Oncology were also searched. All references from relevant publications were scanned manually to identify additional candidate studies. Any MM RCT studying the treatment regimens of induction or ASCT was eligible for our analysis. We extracted data on treatments used for experimental arm and control arm, year of publication, funding type, and number of patients enrolled. For funding type, we categorized the funding source into the broad categories of public, private for profit, or private not for profit. If the funding source were a mix of these categories, funding type was recorded as mixed. For publications that did not report a funding source, we labeled their funding type as unclear.

### Social networks

A social network is represented as a set of nodes denoting different entities such as individuals or organizations and their interactions denoted as ties [10], [11]. SNA is the theoretical framework developed to understand social networks and to reveal hidden and potentially useful information regarding entities and their interactions. SNA has been used extensively to explain phenomena such as (among others) scientific interaction [12], information exchange [13], and treatment success [14]. In this paper, we use SNA to study the RCTs of MM universe focusing on the treatment regimen interactions as they appear in each RCT. Such modeling can provide an in-depth understanding of how treatments and comparisons are distributed and identify the factors associated with development of research questions. [10] We define a treatment network as the set of nodes denoting each treatment tested in an RCT connected with ties denoting a direct comparison between treatments in RCTs (Figure S1 in File S1). The direction of a tie in the treatment network is used to distinguish treatments as experimental and as control. That is, the ties are directed away from the experimental node and toward the control node. Using SNA we can measure the properties of the entire treatment network as well as the properties of each node individually. [15] These measures help us understand how nodes interact and identify opportunities and constraints for future research.

### Node-level properties

Not all nodes in a network are of the same importance. Nodes with a certain position in the network can interact easier/harder with particular nodes and faster/slower with others. To measure the ability of the node to interact with other nodes in the network or to facilitate interactions between nodes we use the *centrality* measures of *degree*, *closeness*, and *betweenness*. In general, centrality measures describe key attributes of the position of a node representing a treatment regimen in the treatment network. [10] Degree measures the number of different comparisons a treatment regimen has participated in; closeness measures the ease at which a treatment regimen can reach other treatment regimens in a network [16], and it is directly associated with the ability to extract information about regimen superiority outside an RCT environment (i.e. indirect meta-analyses); betweenness [17] represents the ability of a treatment regimen to link other treatment regimens, a property that is particularly desirable in indirect meta-analyses. Degree and closeness are further classified into *in*- and *out-degree* and *in*- and *out-closeness* respectively. In the RCT network, in- and out-degree show the number of times a particular regimen has been participating in an RCT as control or experimental respectively. In-closeness shows the easiness at which a node can be reached and out-closeness the easiness at which a node can reach others in the network. When measuring degree and closeness we need to differentiate between control and experimental treatments. Therefore, degree and closeness calculations are performed on the directed treatment networks. On the other hand, when measuring betweenness, we are interested only in the position of the node. Therefore, betweenness calculations were performed on the undirected networks. Note that directed and undirected networks have the same structure (i.e. same number of nodes and number of ties); however, directed networks are represented with directional ties, while undirected networks are represented with bidirectional ties.

### Network level properties

Network level properties are associated with the topology of the entire treatment network. We compute measures such as the network's *density*, *maximum geodesic distance*, *clustering coefficient*, *centrality measures*, *KeyPlayer function*, and the *Girvan-Newman Algorithm* as indicators of the network structure. [10] Density reports the actual treatment interactions in the network as a fraction of all possible interactions (e.g. each regimen is compared to all other regimens). A density value of 15% to 25% indicates that a very small portion of all available comparisons have been achieved and that any changes to the interactions between treatments would have a profound effect on the cohesion of the network. [10] Conversely, values greater than 50% demonstrate that a significant number of comparisons have been made and that changes to interactions will have no or little effect on the cohesion of the network. The maximum geodesic distance corresponds to the network's diameter and it measures the maximum distance between any two treatments in the network. The clustering coefficient is the degree at which treatments in the network form clusters. [10] The clustering coefficient takes values between 0 and 1 where 1 indicates a fully connected network (e.g. each treatment is connected to all the rest). Smaller values of clustering coefficient reveal a rather random pattern of connectivity. [10] The network level centralization measures such as closeness, betweenness, and degree provide an overall impression of network centrality. Interpretation of the network level centralization values is similar to their node level centrality counterparts. For example, a network with low betweenness does not facilitate sharing of information. The Girvan-Newman Algorithm identifies *communities* of treatment regimens. Communities are loosely connected groups of tightly interconnected nodes. [18] The existence of multiple communities may be an indicator of research in isolation. The KeyPlayer function determines the optimal set of treatments in the sense that if an optimal set of nodes were to be removed from the network, this would have a crippling effect. Additionally, the KeyPlayer function determines the most well-connected treatment regimens. Changes to these regimens can influence other treatment regimens. [19]

### 2-mode networks

In addition to the treatment network described in the previous subsections we formed and studied a different category of networks. 2-Mode networks are used to report and analyze the relationships between two different classes of entities. While the treatment network presented previously is used to analyze the relationships between all treatment regimens, the 2-mode network is built to analyze the relationships between treatment regimens and an additional entity such as the funding source or the publication year. We have generated two 2-mode networks. The first 2-mode network (treatment – year) provides a visual association of treatment regimens and the decade they have been used. Similarly, the second 2-mode network (treatment –funding) provides a representation of the funding mechanisms used for each regimen. In the 2-mode networks, the direction of the ties is formed from the experimental and control nodes towards either year of publication or funding type.

### Data analysis

The main analysis included calculation of the following measures: *betweenness*, *density*, *clustering coefficient*, *closeness*, *degree*, *Girvan-Newman Algorithm*, *KeyPlayer*, *maximum geodesic distance* and *distance* among treatments. For the analysis of the node-level and network level properties, we included only treatment regimens from the connected component of the treatment network (e.g. disregarded isolated sets of nodes). The first well-designed RCT of ASCT versus chemotherapy was published in 1996. [20] In order to examine the effect of the first important breakthrough (ASCT) on the evolution of treatment regimens, we also conducted a subgroup analysis of trials published in 1996 or later. Additionally, a time series analysis of the networks with trials from 1996 to 2012 was conducted to compare network properties through time. All networks ware analyzed with UCINET 6 [21] and KeyPlayer 1.44 [19]. Visual representations of the networks were created with NetDraw 2.119. [21]

## Results

### Study selection

**Figure 1:**
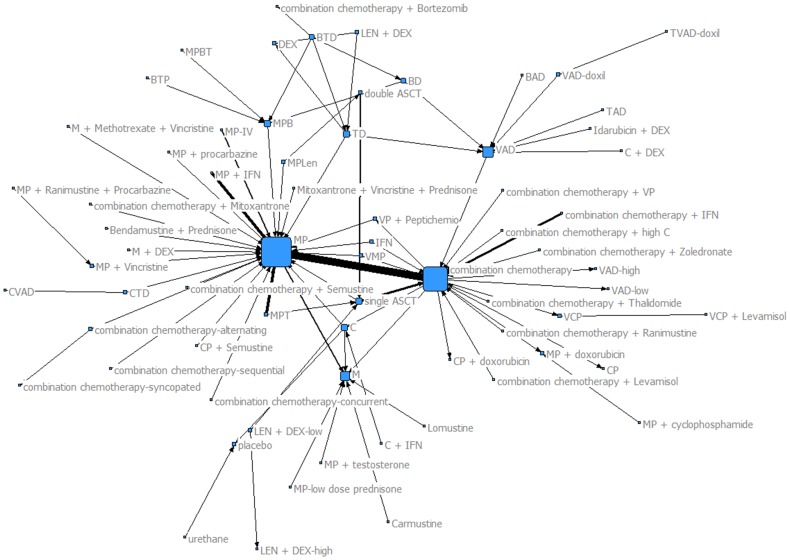
Connected component of RCT treatment network. Each node is associated with a treatment tested in an RCT and each tie denotes a comparison between two treatments. The width of the ties among treatments denotes the number of times two treatments have been tested in RCTs. The network represents the connected component of the network depicted in Figure S1 in File S1 and it is comprised of 155 treatment comparisons. The most frequently tested comparison is between *combination therapy* and *MP*.

We found 135 trials which examined induction or ASCT therapies among patients with MM. Because of multi-arm RCTs, these 135 trials represented 165 comparisons and enrolled 36,869 patients. The entire treatment network is shown in Figure S1 in File S1. Figure 1 depicts the connected portion (connected component) of the entire treatment network that includes 155 comparisons and disregards the unconnected regimens. The treatment regimen abbreviations are listed in Table 1.

**Table 1:** Abbreviations of Treatment Regimens.

Abbreviation	Name of Treatment Regimens
M	melphalan
P	prednisone
C	cyclophosphamide
V	vincristine
MP	melphalan, prednisone
ASCT	autologous stem cell transplant
LEN	lenalidomide
DEX	dexamethasone
TD	thalidomide, dexamethasone
MPT	melphalan, prednisone, thalidomide
MPB	melphalan, prednisone, bortezomib
MPLen	melphalan, prednisone, lenalidomide
BTP	bortezomib, thalidomide, prednisone
BD	bortezomib, dexamethasone
VAD	vincristine, doxorubicin, dexamethasone
MPBT	melphalan, prednisone, bortezomib, thalidomide
TAD	thalidomide, doxorubicin, dexamethasone
TVAD	thalidomide, vincristine, doxorubicin, dexamethasone
BTD	bortezomib, thalidomide, dexamethasone
CTD	cyclophosphamide, thalidomide, dexamethasone
VP	vincristine, prednisone
Q	quinine
IFN	interferon
Combination chemotherapy	vincristine, doxorubicin, melphalan, cyclophosphamide, prednisone
	vincristine, BCNU, doxorubicin, melphalan, prednisone
	vincristine, BCNU, melphalan, cyclophosphamide, prednisone
	vincristine, BCNU, doxorubicin, dexamethasone
	BCNU, doxorubicin, melphalan, cyclophosphamide
	BCNU, melphalan, cyclophosphamide, prednisone
	vincristine, BCNU, doxorubicin, prednisone
	vincristine, doxorubicin, cyclophosphamide, prednisone

### Node-level properties

Table 2 shows selected node-level properties for treatment regimens in the network depicted in Figure 1. The tie thickness, in Figure 1, denotes the frequency that two trials have been compared in an RCT. For example, the treatment regimen of *combination chemotherapy* was frequently compared to the treatment regimen of *MP*. The size of a node represents the number of different comparisons a regimen has been included in (node degree). The larger the size of the node, the greater the number of trials the associated treatment was compared to. The treatment participated in most trials was *MP* followed by *combination chemotherapy*. Also, based on the measures for in- and out-degree we see that MP (in-degree of 72) and combination chemotherapy (in-degree of 23) are the regimens used most often as controls in RCTs. Combination chemotherapy has also been frequently used as experimental therapy in RCTs (out-degree of 39).

**Table 2:** Node-level properties of RCT treatment network (connected component).

Treatment	Betweenness	In-Closeness	Out-Closeness	In-Degree	Out-Degree
MP	1399.8	4.572	1.471	72	4
Combination chemotherapy	1129.5	2.209	1.753	23	39
VAD	451.3	1.694	1.779	7	1
TD	264.3	1.493	1.838	3	4
MPB	207.167	1.538	1.492	4	1
C	197	2.293	1.515	2	4
Single ASCT	188.5	1.538	1.779	7	9
M	262.0	5.732	1.449	11	0
VAD-doxil	67	1.471	1.805	1	1

Regarding closeness (measured using directed network), all nodes had small, relatively similar closeness measures demonstrating a large distance between each node in the network and all other nodes in the network (Table 2). The node-level betweenness (measured using undirected network) was largest for the treatment regimens of *combination chemotherapy, MP*, and *VAD*, indicating that these regimens and those directly connected to them are excellent candidates for indirect meta-analysis.

Distances between selected treatment regimens (undirected network) are reported in Table 3. The majority of the distance between many important treatment regimens (e.g. *MP* to *combination chemotherapy*; *MPT* to *MPB*; *MPT* to *MPLen*; *MPB* to *MPLen*; and *MPB* to *single ASCT)* is equal to 2. This demonstrates that these treatment regimens can reach each other in two “steps”, a property of significance in indirect meta-analyses. This also indicates that these treatment regimens have never been tested in a head-to-head comparison in an RCT.

**Table 3:** Distances between selected treatment regimens.

Measure	Value
Distance from:	
MPT to combination chemotherapy	2
MPB to combination chemotherapy	2
MPLen to combination chemotherapy	2
MPT to MPB	2
MPT to MPLen	2
MPB to MPLen	2
MPT to single ASCT	1
MPB to single ASCT	2
MPLen to single ASCT	2

### Network level properties

The network level properties of *density*, *geodesic distance*, *clustering coefficient*, and the *centrality* measures are reported in Table 4. The density of the network was low, indicating there is little cohesion among the treatments studied. The maximum geodesic distance was equal to 6, indicating that all connected treatments could reach each other in six “steps” within the same pathway of development. All centrality measures are much lower than a similar random network (e.g. Erdos-Renyi with same number of nodes and same density). This shows that the RCT network is decentralized.

**Table 4:** Results of network level analysis for myeloma treatment network.

Measure	RCT network	Erdos-Renyi (random network) value
Density (%)	2.5	2.5
Average Geodesic Distance	3	4.33
Maximum Geodesic Distance	6	10
Clustering Coefficient	3.4	0.025
Network Centralization Metrics		
Betweenness	55.3	78
in-Closeness	1.69	24.1
out-Closeness	1.59	24.1
in-Degree	2.36	4.2
out-Degree	2.13	4.2

**Figure 2:**
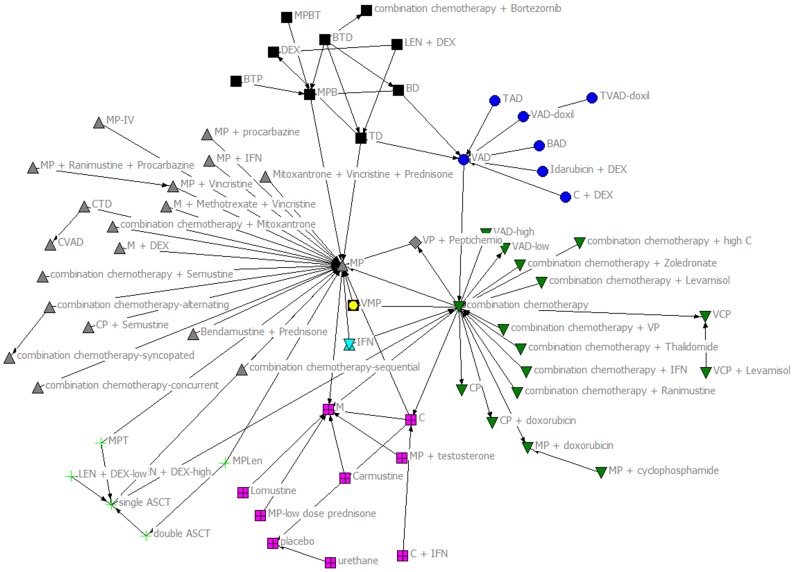
Girvan-Newman Algorithm. The algorithm identified 9 research communities that are loosely connected with each other.

The KeyPlayer function identified that the treatment regimens of *MP*, *combination chemotherapy*, and *VAD* are vital to the network. The Girvan-Newman Algorithm (Figure 2) identified 9 research communities that are loosely connected with each other. These communities would be completely disconnected if the nodes: *MP*, *combination chemotherapy*, and *VAD* were to be removed.

**Figure 3:**
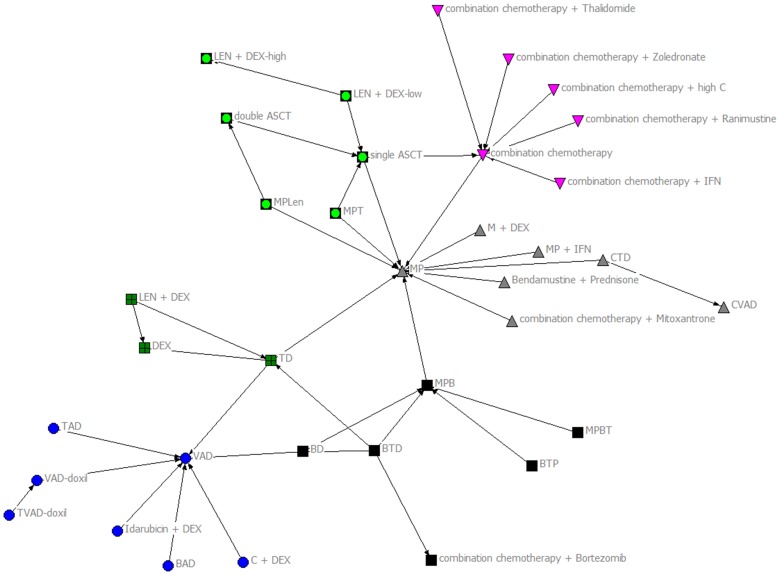
Girvan-Newman Algorithm of RCTs published 1996–2012. The algorithm 6 research communities that are loosely connected with each other.

For our subgroup analysis, we focused on trials published in 1996 or later to determine the effect of the publication by Attal et al., [20] on evolution of treatment regimens. The associated network is shown in Figure S2 in File S1. The KeyPlayer function identified again the *combination chemotherapy*, *MP*, and *VAD* as vital to the network. As in the complete treatment network, the Girvan-Newman Algorithm (Figure 3) shows that there are six research communities that are loosely connected to each other. A visual inspection of Figure 3 shows that these communities would be completely disconnected if the MP, combinational chemotherapy, TD and VAD therapies were to be removed.

### 2-mode network

**Figure 4:**
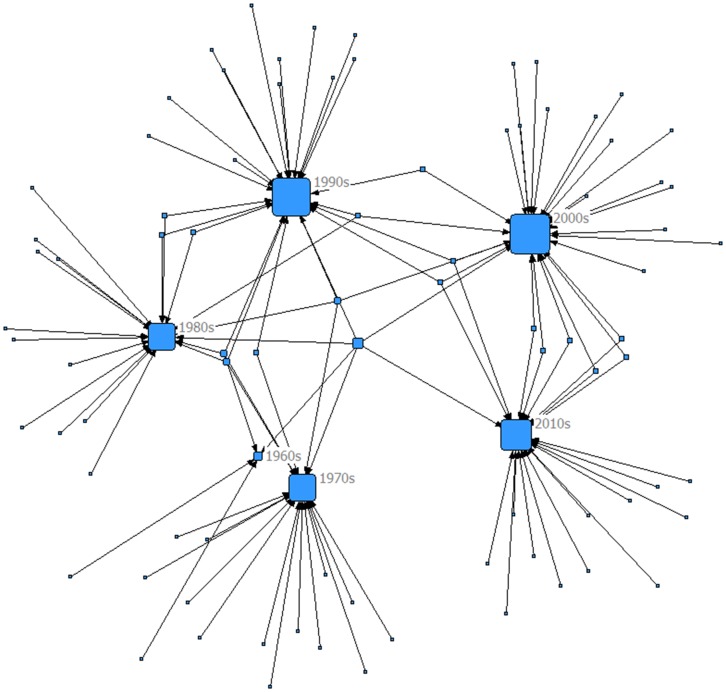
Decades in which treatments have been tested. Most treatment comparisons have been implemented in the decades of the 1990s and 2000s.

Figure 4 demonstrates trends in treatment regimens over time and Figure S4 in File S1 displays the treatment comparisons by funding source. In these figures, degree is represented by the size of the node i.e. the larger the node the more comparisons are directed toward that node. All treatment comparisons were used in these networks, which included 165 comparisons.

In Figure 4, the largest number of treatment regimens was studied in the decades of the 1990s and 2000s, which corresponds to these nodes being the largest out of the six decades in which RCTs of MM patients have existed. As shown in Figure S4 in File S1, the highest number of comparisons came from trials which were publicly funded while the fewest trials were funded by private for profit funding organizations. Out of the 47 trials which did not report funding source, the majority (77%) were trials reported in either abstracts only (full publication not available) at the time this search was conducted or in trials published prior to 1996 when the CONSORT statement [22] was published.

**Figure 5:**
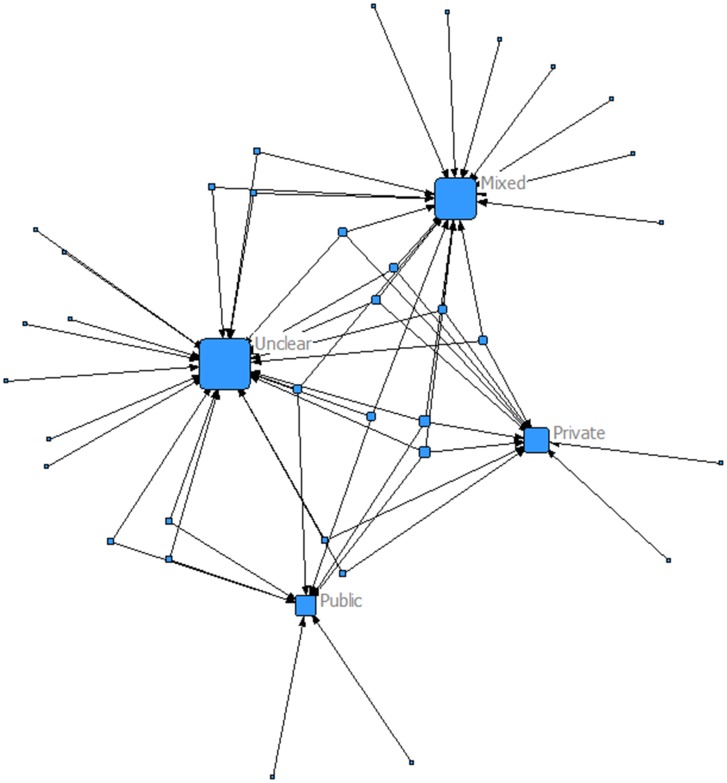
Funding type of trials published in the period 1996–2012. Most treatment comparisons have been funded by a mix of public and private entities. Even though the evidence is missing, we believe that the trials within the unclear funding node have been funded by private entities.

**Figure 6:**
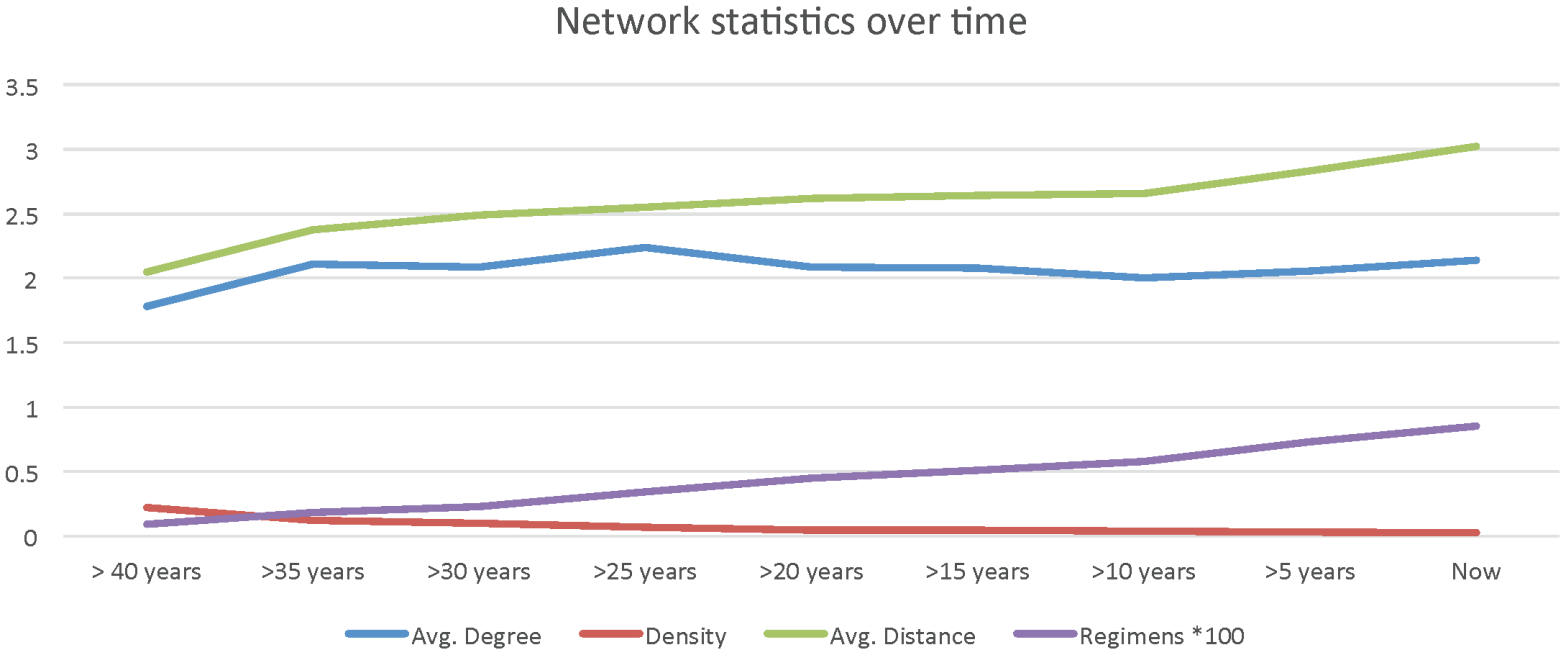
Evolution of centrality measures over time. There is little change in the network's centrality which stresses the fact that there is no coordinated research effort in MM.

The funding trends have changed for RCTs conducted after 1996. Figure 5 demonstrates the results of our subgroup analysis, where it is clear that the majority of RCTs are funded by both public and private entities (mixed). However, there are many trials that did not clearly report their funding mechanisms. The number of these trials is larger than the trials with mixed funding (Figure 6). Even though the evidence is missing, we believe that these trials have been funded by private entities.

Finally, Figure 6 presents the evolution of different centrality measures as the treatment network is constructed over time (Figure S3 in File S1). It is interesting to note that even with the addition of multiple treatment regimens over time, there is very little change in the network's centrality, which stresses the fact that there is no coordinated research effort in MM.

## Discussion

We have analyzed a diverse network of induction and transplant treatment regimens compared in RCTs. Our main finding is that new therapies are compared against established treatments over and over again and not proceeding within a logical framework of testing the most relevant hypotheses. This is demonstrated by the low closeness at both the node and network level and the high betweenness for *MP*, *combination chemotherapy*, *VAD*, and *single ASCT* that do not fully extend to novel agents such as bortezomib, lenalidomide, and thalidomide. This is further exemplified by the fact that there are no direct comparisons between many new regimens, which draws future research directions for RCTs in indirect meta-analysis.

One surprising finding was that through the KeyPlayer function and Girvan-Newman Algorithm we identified two newer treatment regimens, *TD* and *MPB*, which in addition to *combination chemotherapy* and *VAD*, are crucial in maintaining the network structure. However, lenalidomide was not identified as being important in the network even though it is widely used in contemporary practice as first line treatment. These findings point to the discrepancy in regimens being tested in RCTs versus regimens used in practice by the treating Oncologists. The results also demonstrate that researchers tend to compare experimental treatments to inferior regimens. That is, researchers do not compare the most active experimental treatments in head to head trials.

The results of our subgroup analyses show that since 1996 the MM treatment network has evolved in such a way that the novel agents of thalidomide and bortezomib have become very important in maintaining network cohesion. In 1996, Attal et al. [20] demonstrated that ASCT was superior to chemotherapy. This is considered to be the first important breakthrough in the treatment of MM. This subgroup analysis also indicates that chemotherapy and MP are of mostly historical importance.

We also found that the largest number of treatment regimens was studied in the decades of the 1990s and 2000s. The three novel agents of *thalidomide*, *bortezomib*, and *lenalidomide* were developed in the late 1990s and early 2000s which may explain this increase in comparisons of treatment regimens during this time. Additionally, funding source has changed in recent years (since 1996) from being predominately publically funded to a mix of both private and public funding sources.

In this treatment network, designing trials which compare these treatment regimens to each other directly has been avoided by researchers and funders. As a result, better treatment regimens may not be discovered. The fastest rate of discovery occurs when a few hypotheses are tested sequentially. The lack of attention to the entire network is likely the reason that there is current confusion in the field and as a result guideline panels [23] are not able to provide conclusive recommendations on the best first-line treatment from a list of 83 regimens.

Our analysis has some limitations. The main limitation is that we are inferring reasons from published trials on why researchers and funders choose which treatment to study. Since we only included trials examining treatment regimens used in induction or transplant setting, we cannot decipher how this treatment network fits into the larger network of all MM trials. Future research should focus on conducting a SNA on other areas of MM treatment such as supportive, maintenance, and salvage therapies. Nevertheless, our network analysis focuses on giving insight into first-line treatment, which is the practitioner's best attempt to slow down the progression of disease.

Furthermore, the current design does not allow for analysis of single arm RCTs, which may provide valuable information regarding a regimen's efficacy in MM, and does not include phase I or phase II trials. Finally, since this is the first SNA study on RCT treatments, it is impossible to compare our findings with similar networks in different diseases, which forced us to use random networks for comparison.

Performing SNA in RCTs may provide both funders and researchers with the overall assessment of existing evidence in a given field within the totality of research efforts that may help avoid isolation and duplication. Once performed, researchers may visualize the overall research network and determine the relevance of their hypotheses and if necessary derive future research directions such as those suggested in this study (e.g. MP to combination chemotherapy; MPT to MPB; MPT to MPLen; MPB to MPLen; and MPB to single ASCT).

We conclude that research programs in myeloma are decentralized with a lack of connectivity among various research pathways. This results in lack of head-to-head RCTs of novel agents compared to each other or single ASCT. We have demonstrated that by using SNA to visually and analytically examine treatment networks prior to designing a clinical trial, researchers can better design studies to address more relevant clinical and research questions.

## Supporting Information

File S1 Contains the following files: **Figure S1.** Entire treatment network. Each node is associated with a treatment participating in an RCT and each tie denotes a comparison between two treatments. The width of the ties among treatments denotes the number of times two treatments have been tested in RCTs. The network is comprised of 165 treatment comparisons. **Figure S2.** Connected component of network of RCTs published between1996–2012. The width of the ties among treatments denotes the number of times two treatments have been tested in RCTs. **Figure S3.** Evolution of the treatment network over time. The figure presents the network's topology 40, 30, 20 and 1 year ago. **3a.** RCT network that includes trials performed more than 40 years ago; **3b.** RCT network that includes trials performed more than 30 years ago; **3c.** RCT network that includes trials performed more than 20 years ago; **3d.** RCT network that includes trials performed more than 1 year ago. **Figure S4** Funding type of all trials. Most treatment comparisons have been funded by the public sector.
(DOCX)
