# Supplementary material for: Evolution of Treatment Regimens in Multiple Myeloma: A Social Network Analysis
Source: PLoS One. 2014 Aug 13;9(8):e104555. doi: 10.1371/journal.pone.0104555 (PMC4131914; doi:10.1371/journal.pone.0104555)
Supplement: File S1 — Contains the following files: Figure S1. Entire treatment network. Each node is associated with a treatment participating in an RCT and each tie denotes a comparison between two treatments. The width of the ties among treatments denotes the number of times two treatments have been tested in RCTs. The network is comprised of 165 treatment comparisons. Figure S2. Connected component of network of RCTs published between1996–2012. The width of the ties among treatments denotes the number of times two treatments have been tested in RCTs. Figure S3. Evolution of the treatment network over time. The figure presents the network's topology 40, 30, 20 and 1 year ago. 3a. RCT network that includes trials performed more than 40 years ago; 3b. RCT network that includes trials performed more than 30 years ago; 3c. RCT network that includes trials performed more than 20 years ago; 3d. RCT network that includes trials performed more than 1 year ago. Figure S4 Funding type of all trials. Most treatment comparisons have been funded by the public sector. (DOCX) [file pone.0104555.s001.docx]

**Evolution of treatment regimens in multiple myeloma: a social network analysis**

Helen Mahony_­_, Athanasios Tsalatsanis, Ambuj Kumar, Benjamin Djulbegovic

**Supplemental Material**


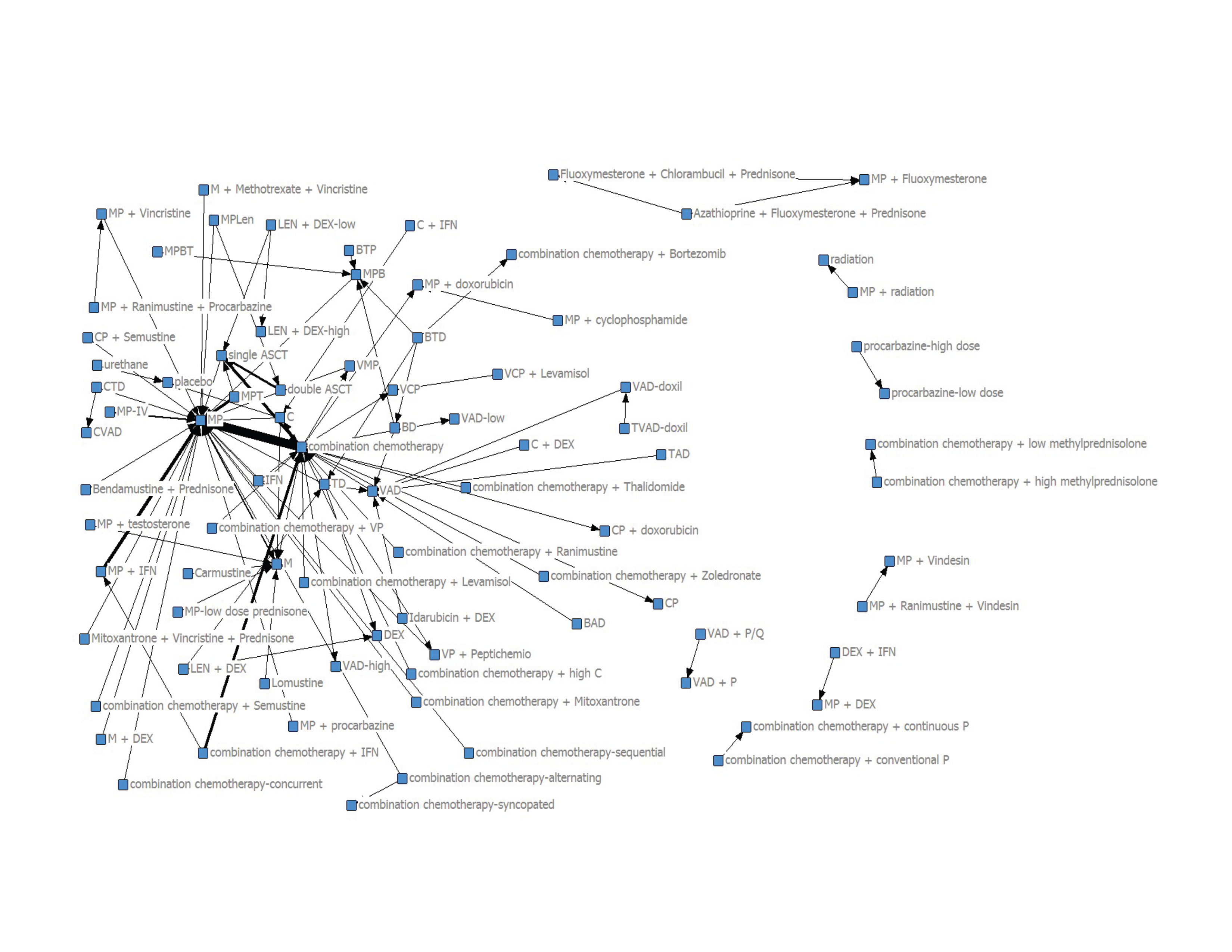


**Figure S1.** Entire treatment network. Each node is associated with a treatment participating in an RCT and each tie denotes a comparison between two treatments. The width of the ties among treatments denotes the number of times two treatments have been tested in RCTs. The network is comprised of 165 treatment comparisons.

**
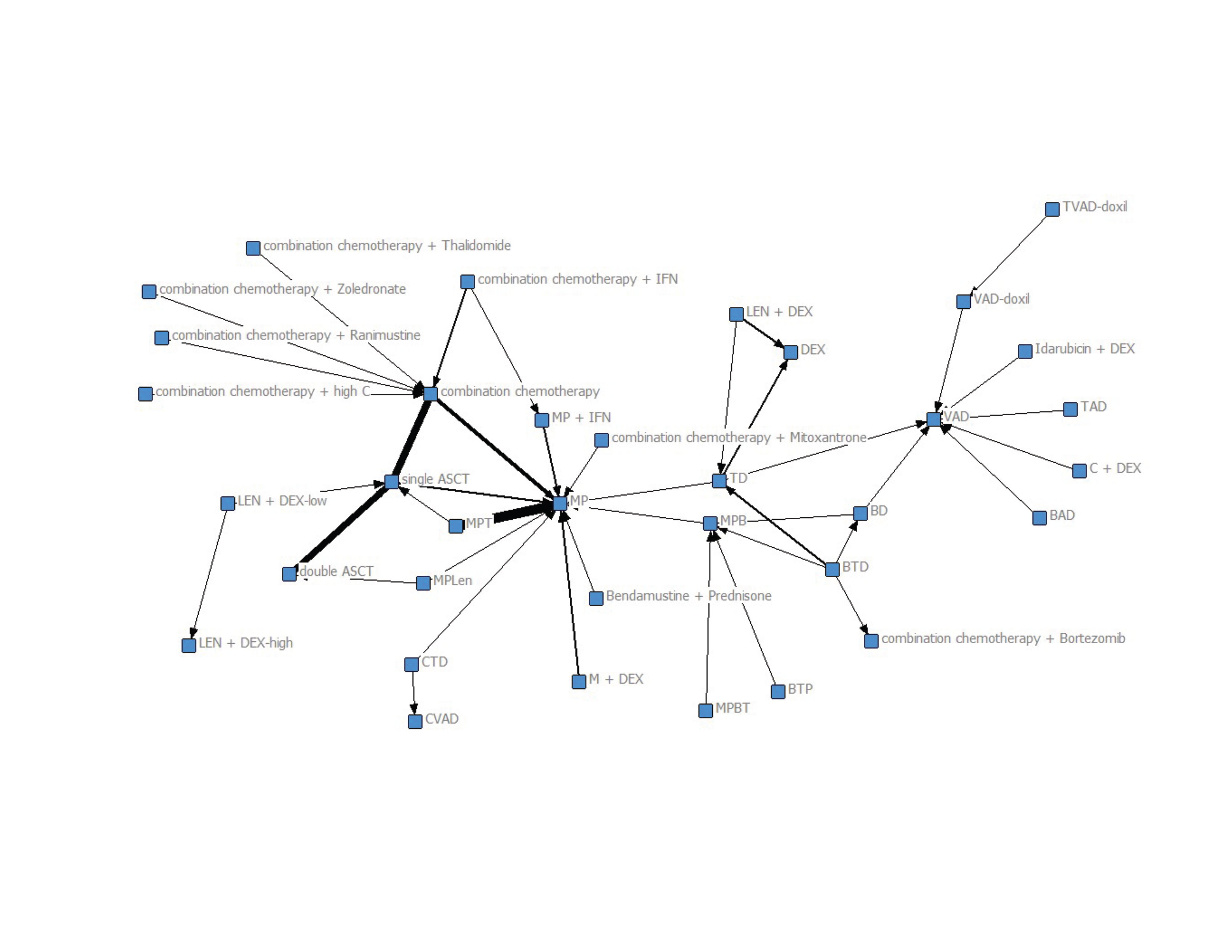
**

**Figure S2.** Connected component of network of RCTs published between1996-2012. The width of the ties among treatments denotes the number of times two treatments have been tested in RCTs.


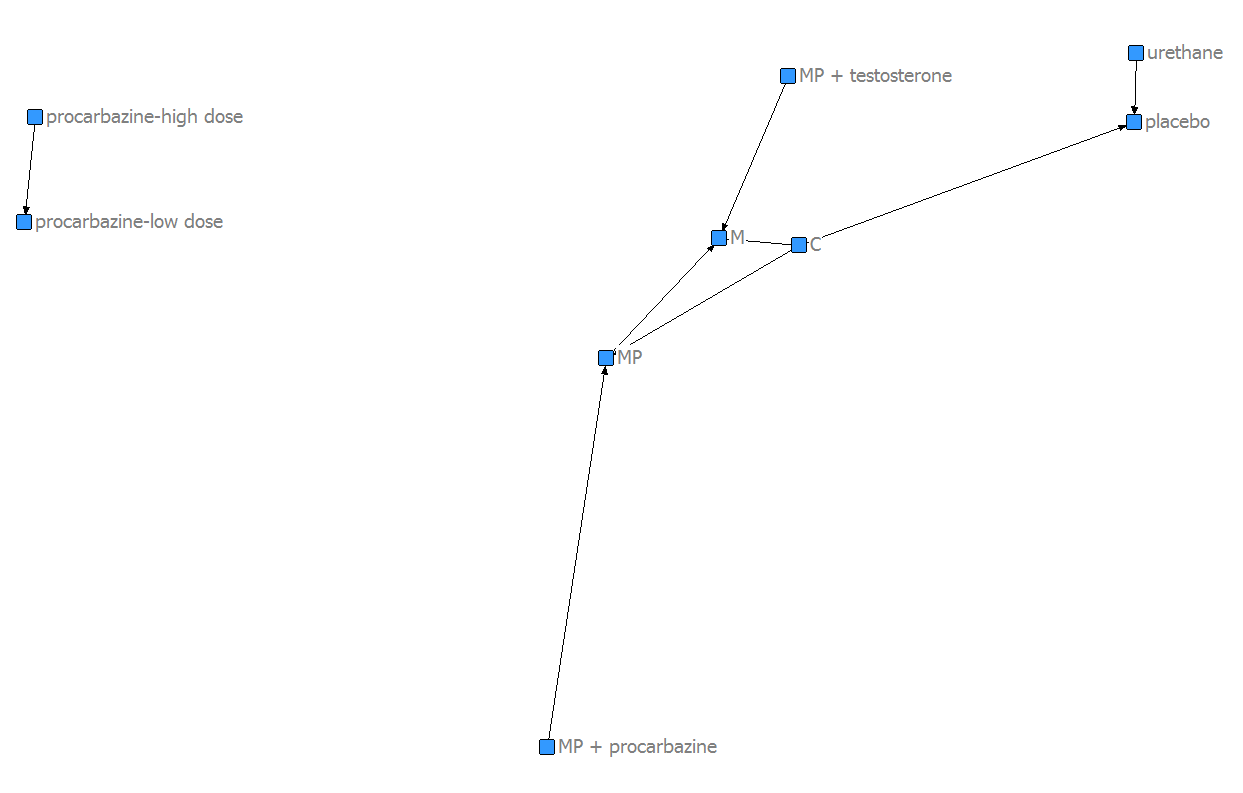
3a. RCT network that includes trials performed more than 40 years ago


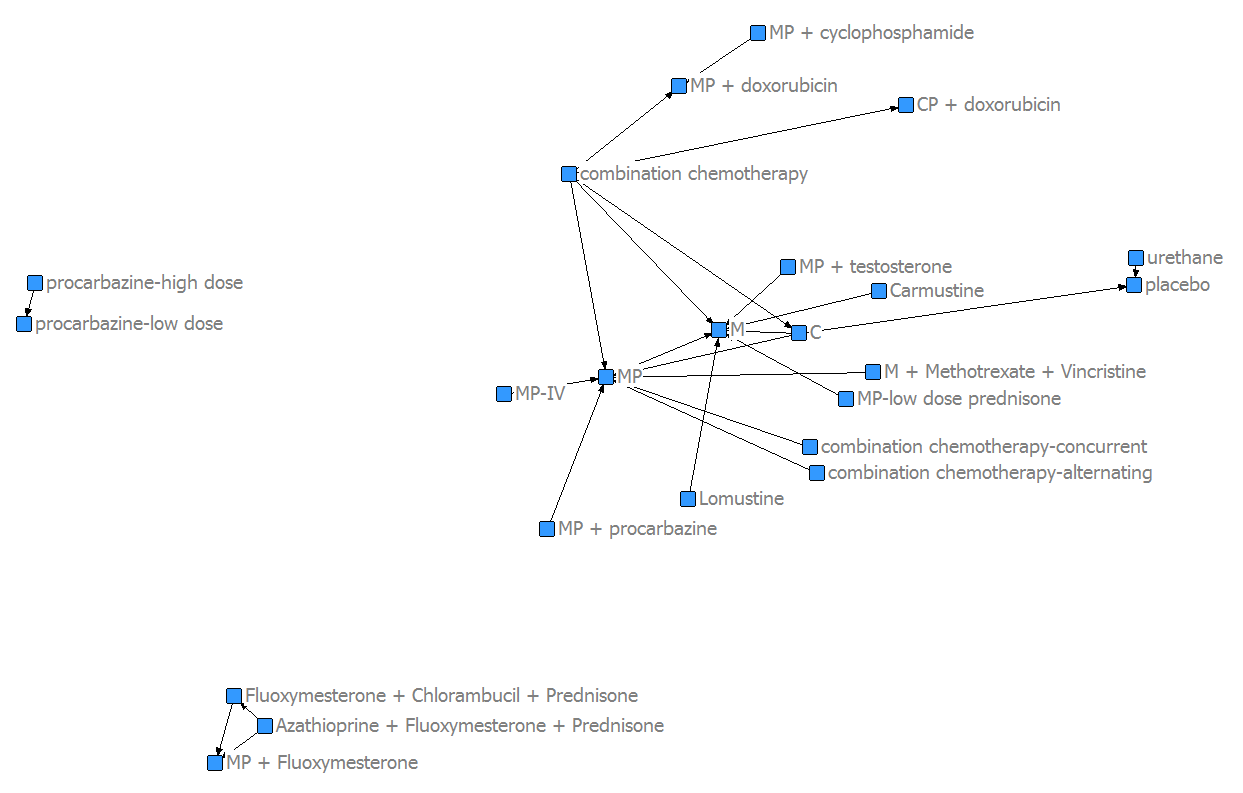


3b. RCT network that includes trials performed more than 30 years ago


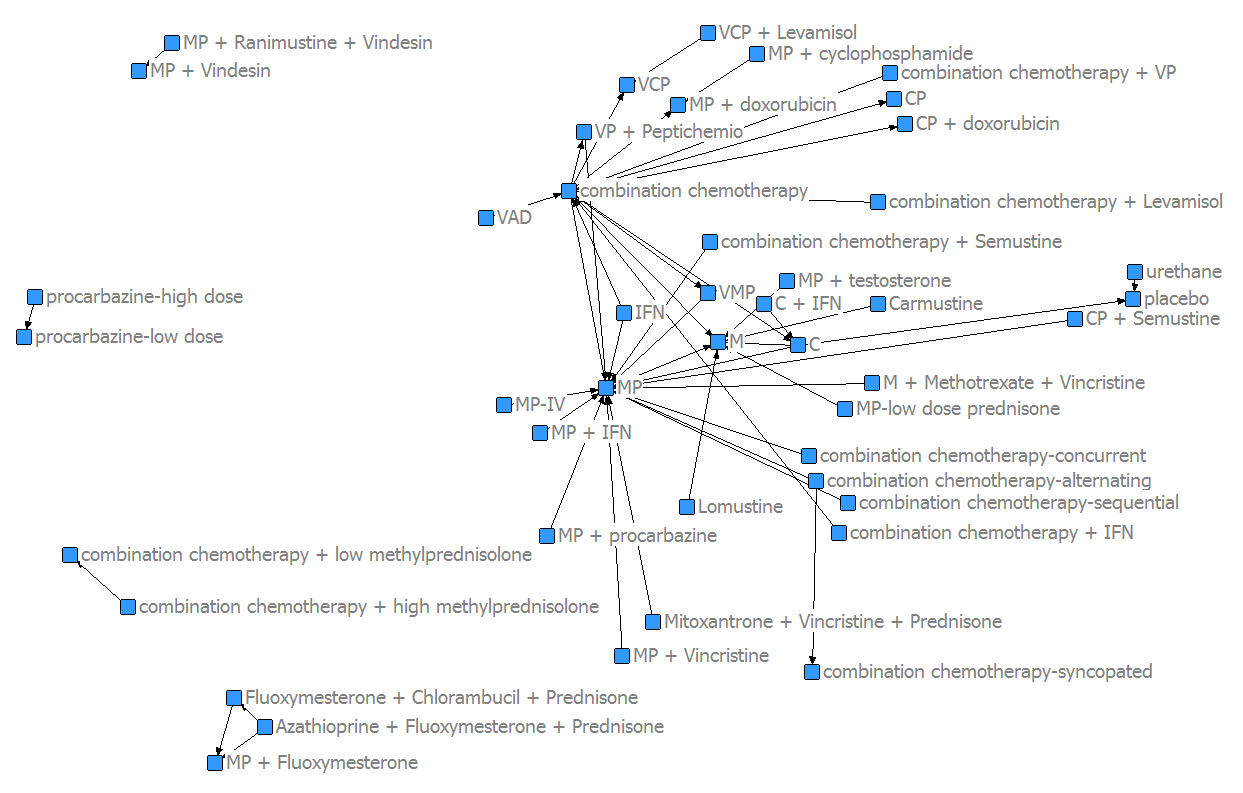


3c. RCT network that includes trials performed more than 20 years ago


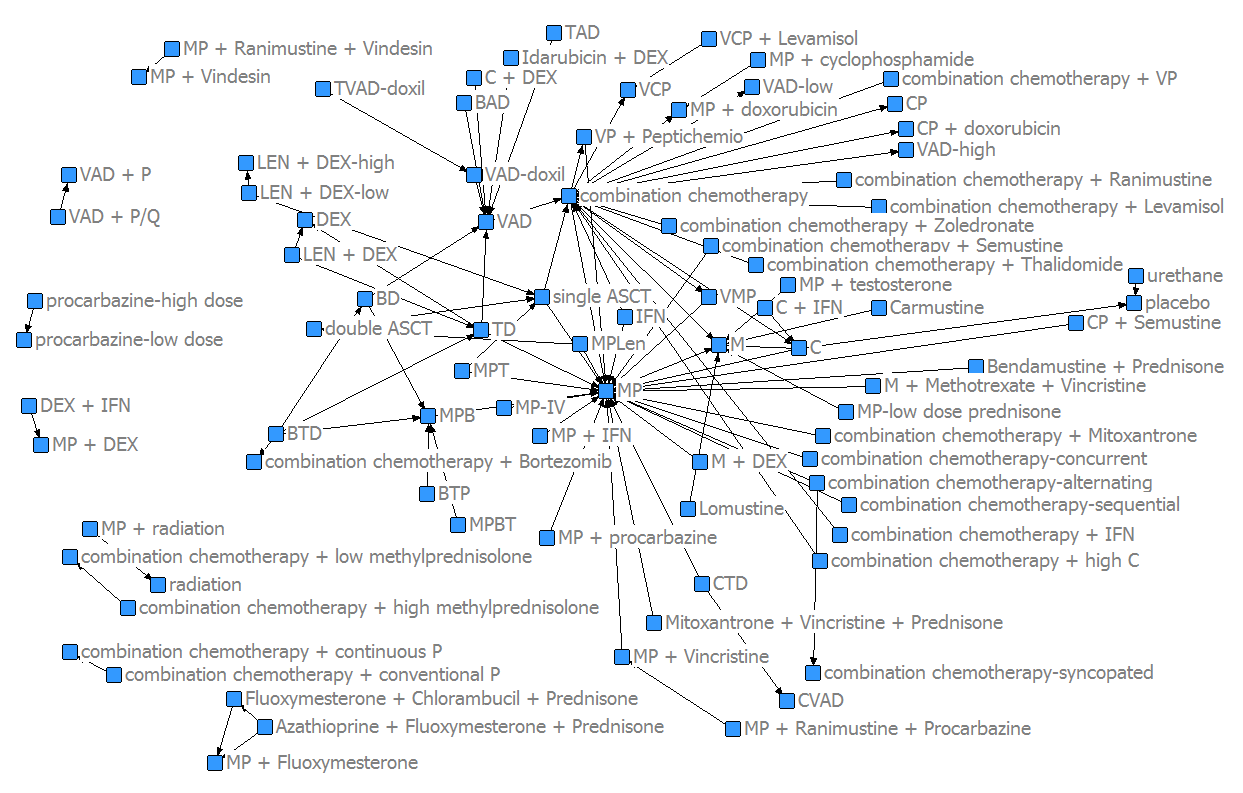


3d. RCT network that includes trials performed more than 1 year ago

**Figure S3.** Evolution of the treatment network over time. The figure presents the network’s topology 40, 30, 20 and 1 year ago.


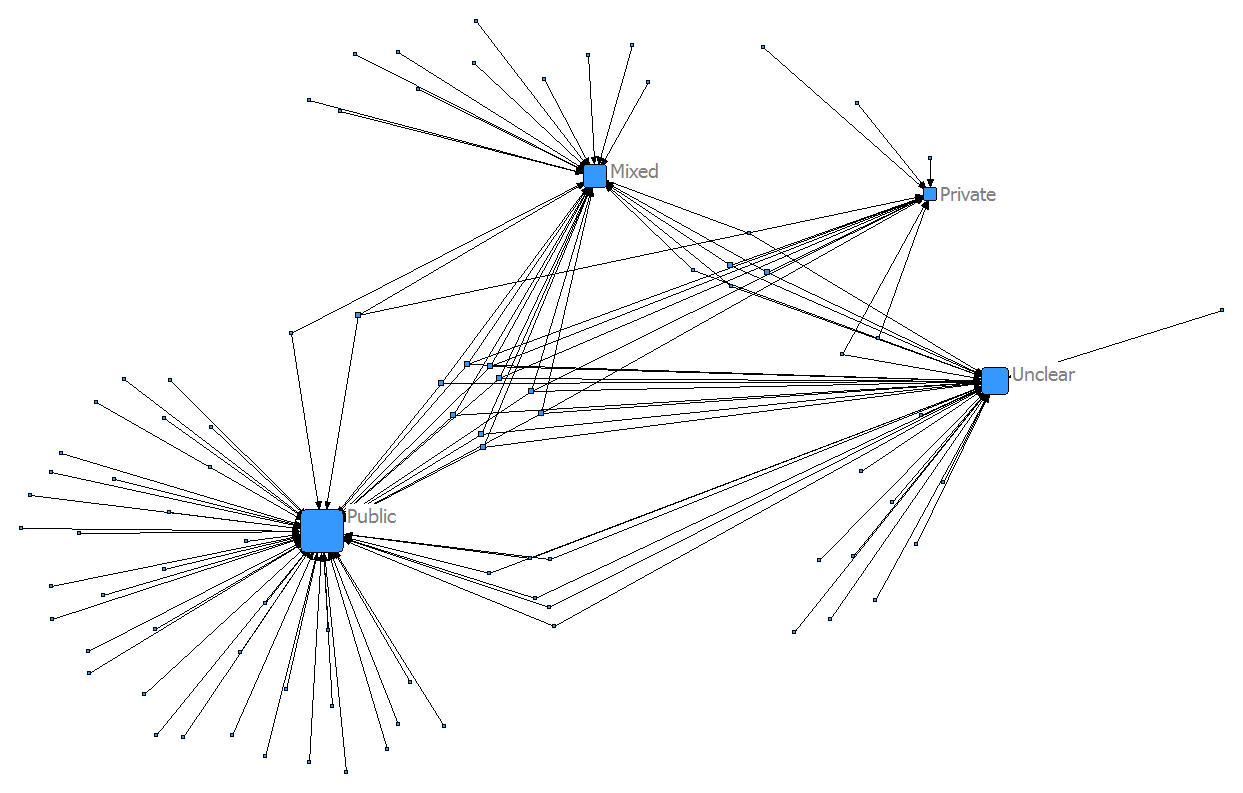


**Figure S4** Funding type of all trials. Most treatment comparisons have been funded by the public sector.
